# First complete genome sequence and comparative analysis of *Salmonella enterica* subsp. *diarizonae* serovar 61:k:1,5,(7) indicates host adaptation traits to sheep

**DOI:** 10.1186/s13099-019-0330-9

**Published:** 2019-10-14

**Authors:** Laura Uelze, Maria Borowiak, Carlus Deneke, Cécile Jacobs, István Szabó, Simon H. Tausch, Burkhard Malorny

**Affiliations:** 1 Bundesinstitut für Risikobewertung (BfR), Max-Dohrn-Str. 8-10, 10589 Berlin, Germany; 2 Landeslabor Schleswig-Holstein, Max-Eyth-Straße 5, 24537 Neumünster, Germany

**Keywords:** *Salmonella enterica* subsp. *diarizonae*, Host-adaptation, Pseudogenes, Sheep

## Abstract

**Background:**

The *Salmonella enterica* subsp. *diarizonae* serovar 61:k:1,5,(7) (SASd) has been found to be host-adapted to sheep, with a high prevalence in sheep herds worldwide. Infections are usually sub-clinical, however the serovar has the potential to cause diarrhea, abortions and chronic proliferative rhinitis. Although occurrence and significance of SASd infections in sheep have been extensively studied, the genetic mechanism underlying this unusual host-adaptation have remained unknown, due to a lack of (a) available high-quality genome sequence(s).

**Results:**

We utilized Nanopore and Illumina sequencing technologies to generate a de novo assembly of the 4.88-Mbp complete genome sequence of the SASd strain 16-SA00356, isolated from the organs of a deceased sheep in 2016. We annotated and analyzed the genome sequence with the aim to gain a deeper understanding of the genome characteristics associated with its pathogenicity and host adaptation to sheep. Overall, we found a number of interesting genomic features such as several prophage regions, a VirB4/D4 plasmid and novel genomic islands. By comparing the genome of 16-SA00356 to other *S. enterica* serovars we found that SASd features an increased number of pseudogenes as well as a high level of genomic rearrangements, both known indicators of host-adaptation.

**Conclusions:**

With this sequence, we provide the first complete and closed genome sequence of a SASd strain. With this study, we provide an important basis for an understanding of the genetic mechanism that underlie pathogenicity and host adaptation of SASd to sheep.

## Background

*Salmonella enterica* subsp. *diarizonae* serovar 61:k:1,5,(7) (also designated as SASd) is a Gram-negative bacterium of the genus *Salmonella*. SASd is considered host-adapted to sheep, based on its wide distribution and high prevalence in sheep flocks worldwide [1–7]. SASd colonizes the intestines and tonsils of sheep and can be isolated from the faeces and nasal discharge of the animals [6]. Colonization might be chronic, with faecal shedding of the pathogen allowing transmission between individuals [8]. Although the serovar does not usually induce diseases [8–10], it has the potential to cause diarrhea [11], abortions [2] and chronic proliferative rhinitis [3, 6]. Over the last years, occurrence, distribution and impact of SASd infections in sheep have been extensively studied. However the potential genetic features underlying this unusual host-adaptation have remained unknown, due to a lack of available high-quality genome sequences. Here, we announce the first, complete and closed genome sequence of *S. enterica* subsp. *diarizonae* serovar 61:k:1,5,(7). Through genome analysis and a genome comparison study we identified numerous genetic features indicating host adaptation traits of SASd to sheep.

## Methods

### Strain isolation and characterization

Strain 16-SA00356 was isolated from an enriched pooled organ sample of an adult sheep that was found dead in Northern Germany in 2016. The sheep was postmortem diagnosed with liver cell necrosis, liver abscesses and serofibrinous peritonitis, most likely resulting from an infection with *Fasciola hepatica*. Detection of *Salmonella* spp. in the enriched culture was considered an incidental finding. Enrichment and isolation of *Salmonella* spp. was achieved by pooling small pieces of different organs (lung, liver, kidney, spleen and small intestine) in tetrathionate broth of Preuss, a selective medium for enrichment of *Salmonella* spp., followed by incubation for 12 h at 37 °C. After incubation, the broth was spread on three selective solid agar plates (Rambach agar, XLD agar, BSB agar), followed by another incubation cycle. *Salmonella* spp. colonies were confirmed through MALDI-TOF, before further subcultivation on Lysine Iron Agar (LIA) slants. The *Salmonella* isolate was serotyped by slide agglutination with the antigenic formula 61:k:1,5,(7). Antimicrobial susceptibility testing was performed by broth microdilution following CLSI guidelines (CLSI M07-A9) and EUCAST epidemiological cut-off values (ECOFFs; http://www.eucast.org/). The isolate was found to be sensitive to all tested antibiotics (ampicillin, chloramphenicol, ciprofloxacin, colistin, cefotaxime, gentamicin, nalidixic acid, sulfamethoxazole, ceftazidime, tetracycline and trimethoprim).

### Genome sequencing and de novo assembly

Genomic DNA for both sequencing techniques was isolated from an overnight liquid culture using the PureLink^®^ Genomic DNA Mini Kit (Invitrogen, Carlsbad, CA, USA). Sequencing libraries for Illumina sequencing were prepared with the Nextera XT DNA Sample Preparation Kit (Illumina, San Diego, CA, USA) according to the manufacturer’s protocol. Sequencing was performed in 2 × 251 cycles on the Illumina MiSeq benchtop using the MiSeq Reagent v3 600-cycle Kit (Illumina). A total number of 1,433,866 reads was generated, with 86% of bases above a quality score of 30 (> Q30) and an overall coverage of 60×. The paired Illumina reads were trimmed using Trimmomatic v0.36 [12] with option *sliding window* 4:20 and *minlen* 50 yielding 1,295,014 read pairs. To generate long reads for scaffolding, Oxford Nanopore MinION technology (ONT) was applied. MinION libraries were prepared using the Rapid barcoding kit (Oxford Nanopore Technologies, Oxford, UK), following the manufacturer’s instructions, and sequenced for approximately 16 hours using a FLO-MIN106 R9 flow cell generating 82,147 reads. The genome was assembled with Unicycler v0.4.4 [13], including Pilon v1.23 [14], providing the trimmed Illumina reads as paired short reads and the ONT reads as long reads with default parameters.

### Genome annotation

Antibiotic resistance genes were identified with ResFinder v3.1 [15]. Salmonella pathogenicity islands (SPI) were detected with SPIFinder v1.0 with default parameters [16] and by BLAST against known Salmonella pathogenicity islands. Prophage regions were identified with PHASTER [15]. Pseudogenes were determined with Pseudofinder v0.10 [17] with standard parameters and *length* 0.8. Genomic rearrangements were detected with progressive Mauve v2.4.0, with standard parameters [18].

### Comparative genomic analysis

The genome of the SASd isolate 16-SA00356 was compared to a set of well annotated *S. enterica* serovars which were chosen to represent different host ranges. All serovars together with their NCBI accession numbers and information regarding the size of their genomes, number of ORFs and GC content, are listed in Additional file 1: Table S1. Plasmid sequences were excluded from the comparative analysis.

### Phylogenetic analysis

Phylogeny was inferred through alignment free genome comparison with feature frequency profiles (FFP v3.19) and through comparison of 107 essential single-copy core genes following the bcgTree pipeline (v1.1.0). The bcgTree pipeline was applied with default parameters as as described by Ankenbrand and Keller [19]. FPP was performed with default parameters and *l*-*mer length* 24 as described by Wang and Ash [20].

### Quality assurance

A single colony of 16-SA00356 was transferred to fresh LB medium to obtain a pure culture for genomic DNA extraction. After the genome sequence was obtained subspecies and serovar assignments were confirmed by the in silico typing tool SISTR v1.0.2 [21].

## Results and discussion

### General features

**Fig. 1:**
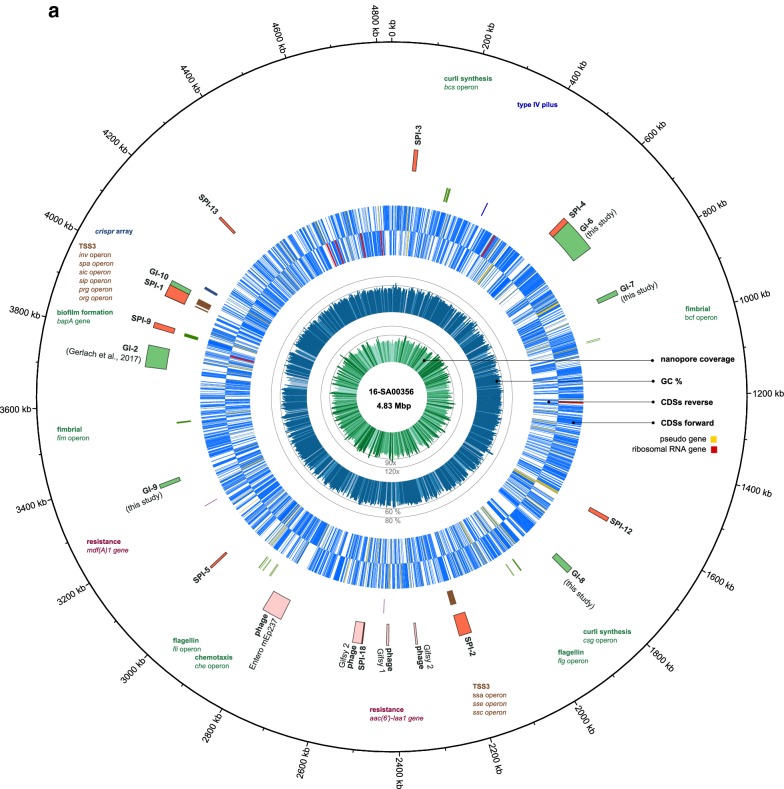

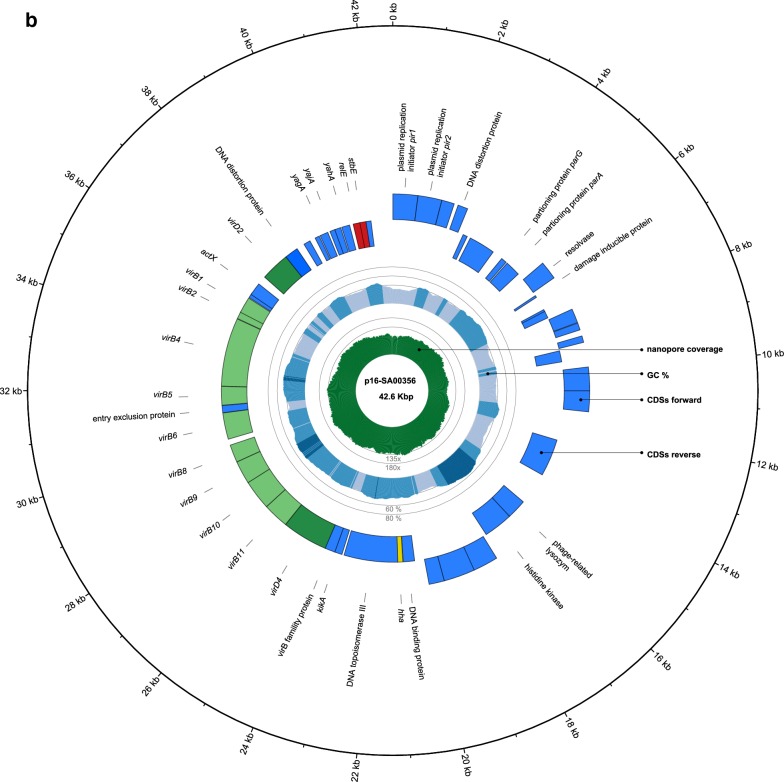
Genome (**a**) and plasmid (**b**) map of *S. enterica* subsp. *diarizonae serovar* 61:k:1,5,(7), isolate 16-SA00356, displayed in Circos. The tracks from inside to outside represent the Nanopore sequencing coverage, GC content, reverse-strand CDSs, forward-strand CDSs and labeled genetic regions of interest such as important operons, Salmonella pathogenicity islands (SPI), genomic islands (GI) and prophage regions

The genome of SASd isolate 16-SA00356 is composed of a circular chromosome of 4,832,672 bp (GC 51.49%) and a circular plasmid of 42,663 bp (GC 41.34%). A graphical representation of the annotated chromosome and plasmid is shown in Fig. 1. A total of 4687 CDSs, 80 tRNAs, 1 tmRNA and 22 rRNAs regions were predicted within the chromosome by the NCBI Prokaryotic Genome Annotation Pipeline (PGAP).

### Antibiotic resistance and pathogenicity

Although the strain was susceptible to eleven tested antibiotics, two antibiotic resistance genes homologous to *aac(6′)*-*Iaa* (Accession: NC_003197), an aminoglycoside acetyltransferase, and *mdfA* (Accession: Y08743), a macrolide–lincosamide–streptogramin B (MLS) resistance gene were identified. We found that the strain possesses the major pathogenicity islands SPI-1, SPI-2, SPI-3, SPI-4, SPI-5, SPI-9, SPI-12, SPI-13, SPI-18 and GI-2 [22]. Furthermore, by comparing the sequence based similarity of the 16-SA00356 genome to five other *S. enterica* subsp. *diarizonae* serovars (see Additional file 2: Figure S1) we identified five novel genomic islands: GI-6 - GI-10. These novel genomic islands contain mainly proteins of unknown function and no major virulence genes could be attributed to them. In addition, we identified four incomplete prophage regions, with high similarity to the prophages Entero mEp237, Gifsy 1 and Gifsy 2. Overall, the resistance and virulence potential of SASd appears to be low. The absence of major antibiotic resistance genes can be attributed to the generally low antibiotic usage/low intensity farming practice of sheep.

### Plasmid

SASd isolate16-SA00356 was found to carry an IncX1/ColRNAI type plasmid of 42,663 bp which we named pSE16-SA00356. We found pSE16-SA00356 to harbour an almost complete conjugative Type IV secretion system (missing *virB7*), which has been linked to persistent infections in numerous pathogens [23]. The plasmid furthermore carries the RelE/StbE toxin/antitoxin system and a small Haemolysin expression-modulating protein Hha, although the complementary *tomB* antitoxin gene was not detected in our analysis. The addiction module RelE/StbE probably increases the stability and therefore the persistence of the plasmid in the microbial population.

### Phylogenetic analysis

**Fig. 2:**
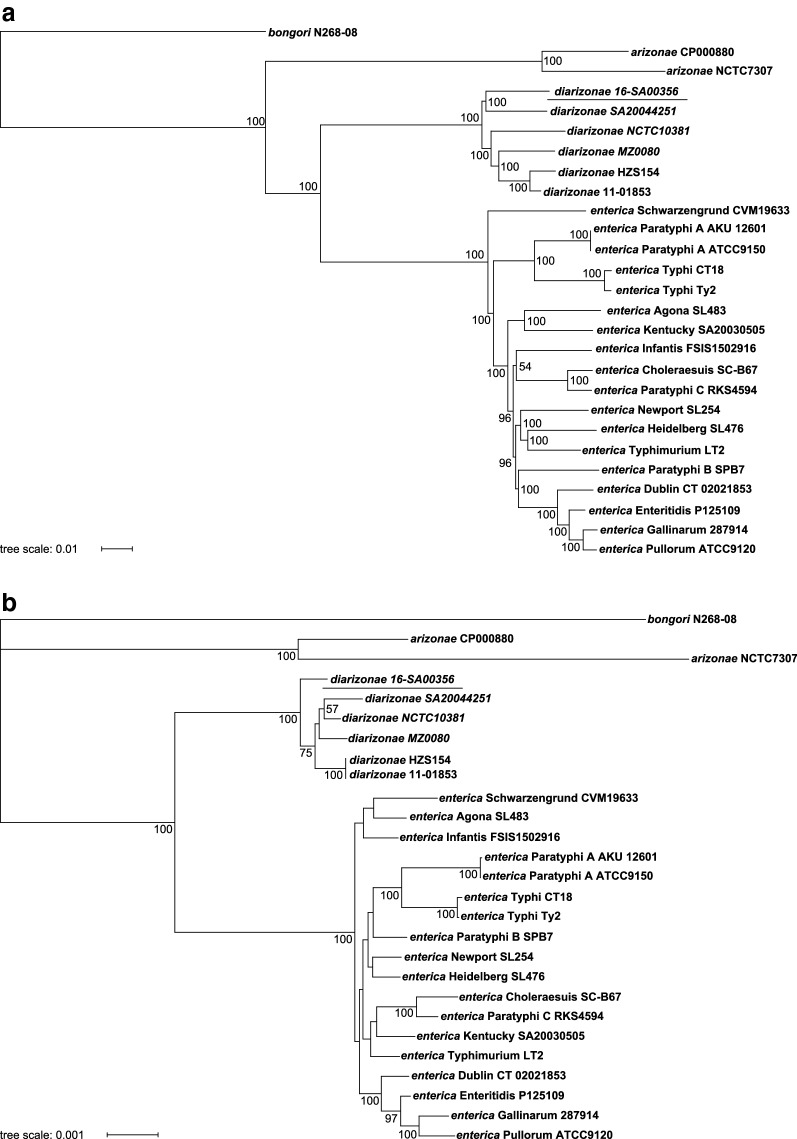
Phylogenetic analysis of *Salmonella* species with *Salmonella bongori* N268-08 (CP006608) as outgroup. **a** NJ tree based on the comparison of complete genome sequences with the alignment-free feature frequency profiles (FFP) method. Numbers at nodes designate bootstrap support values generated using 100 permutations. **b** Best-scoring maximum-likelihood tree based on the comparison of the amino acid sequences of 107 essential single-copy core genes with bcgTree. Numbers at nodes designate bootstrap support values resulting from 100 bootstrap replicates. Only bootstrapping values greater than 50 are displayed

Phylogeny of the different *Salmonella* species was inferred through alignment-free genome comparison with feature frequency profiles (FFP) and through comparison of 107 essential single-copy core genes with bcgTree. The resulting phylogenetic trees are shown in Fig. 2 and both indicate that 16-SA00356 clusters within the group of the *S. enterica* subsp. *diarizonae* serovars. Bootstrap values attribute greater certainty to the pyhlogenetic tree obtained through FFP.

### Pseudogenes

Recent intracellular pathogens have a higher number of pseudogenes that results from the fact that adaptation to an intracellularly lifestyle causes bacteria to gradually loses genes no longer needed in their new environment. Nuccio and Bäumler [24] propose that *Salmonella* serovars could be divided into a group with a low number of pseudogenes and those with a high number of pseudogenes, with the later group referred as the extraintestinal pathovars. When comparing the percentage of pseudogenes normalized to the total number of ORFs among different *Salmonella* serovars we found SASd isolate16-SA00356 to possess a medium number of pseudogenes. An overview of the results of our analysis is shown in Table 1. Overall, host-adapted and host-restricted serovars such as *S. enterica* subsp. *enterica* serovar Choleraesuis (pigs), *S. enterica* subsp. *enterica* serovar Typhi (humans), *S. enterica* subsp. *enterica* serovars Gallinarum and Pullorum (birds) feature a higher percentage of pseudogenes (6.5–7.6%), than those reported to have a broad host range i.e. *S. enterica* subsp. *enterica* serovar Typhimurium (4.9%). Interestingly, the genome of the SASd isolate 16-SA00356 features a comparable number of pseudogenes (6.0%), to the cattle-adapted *S. enterica* subsp. *enterica* serovars Dublin (5.7%) and Kentucky (5.5%). Together with the fact that among the investigated *S. enterica* subsp. *diarizona*e serovars, SASd possesses the highest number of pseudogenes, these results further indicate a host-adaptation to sheep.

**Table 1:** Correlation between number of pseudogenes and host range of the respective organism

Serovar	Host range	ORFs	Pseudogenes	%
*S. enterica* subsp. *enterica* serovar Infantis FSIS1502916	Broad [26]	4407	206	4.67
*S. enterica* subsp. *enterica* serovar Agona SL483	Broad [27]	4444	213	4.79
*S. enterica* subsp. *enterica* serovar Schwarzengrund CVM19633	Broad [27]	4410	212	4.81
*S. enterica* subsp. *enterica* serovar Paratyphi B SPB7	Broad [27]	4549	220	4.84
*S. enterica* subsp. *diarizonae* serovar 60:r:z HZS154	Unknown	4689	228	4.86
*S. enterica* subsp. *enterica* serovar Typhimurium LT2	Broad [27, 28]	4504	220	4.88
*S. enterica* subsp. *enterica* serovar Heidelberg SL476	Broad [27]	4565	223	4.88
*S. enterica* subsp. *diarizonae* serovar 65:c:z SA20044251	Unknown	4461	218	4.89
*S. enterica* subsp. *enterica* serovar Newport SL254	Broad [27]	4489	220	4.90
*S. enterica* subsp. *enterica* serovar Enteritidis P125109	Broad [27]	4352	223	5.12
*S. enterica* subsp. *diarizonae* serovar 61:i:z NCTC10381	Unknown	4828	249	5.16
*S. enterica* subsp. *diarizonae* serovar 50:k:z MZ0080	Unknown	4684	245	5.23
*S. enterica* subsp. *diarizonae* serovar 60:r:z 11-01853	Unknown	4326	227	5.25
*S. enterica* subsp. *enterica* serovar Kentucky SA20030505	Bovine-adapted [28]	4427	242	5.47
*S. enterica* subsp. *enterica* serovar Dublin CT_02021853	Bovine-adapted [27, 28]	4580	261	5.70
*S. enterica* subsp. *diarizonae* serovar 61:k:1,5,(7) 16-SA00356	Sheep-adapted [5]	4461	269	6.03
*S. enterica* subsp. *enterica* serovar Paratyphi C RKS4594	Human-restricted [29]	4615	285	6.18
*S. enterica* subsp. *enterica* serovar Paratyphi A AKU_12601	Human-restricted [27, 28]	4340	281	6.47
*S. enterica* subsp. *enterica* serovar Paratyphi A ATCC 9150	Human-restricted [27, 28]	4337	281	6.48
*S. enterica* subsp. *enterica* serovar Choleraesuis SC-B67	Porcine-adapted [27]	4566	296	6.48
*S. enterica* subsp. *enterica* serovar Typhi CT18	Human-restricted [27, 28]	4665	307	6.58
*S. enterica* subsp. *enterica* serovar Typhi Ty2	Human-restricted [27, 28]	4621	314	6.80
*S. enterica* subsp. *enterica* serovar Pullorum ATCC 9120	Avian-restricted [29, 30]	4474	334	7.47
*S. enterica* subsp. *enterica* serovar Gallinarum 287/91	Avian-restricted [27, 28]	4453	336	7.55

Host ranges were inferred from the literature (when available). The number of ORFs and pseudogenes was determined with Pseudofinder [17]. Only complete genome sequences were considered. Plasmid sequences were excluded from the analysis

### Genome rearrangements

Host restricted pathogens often exhibit genomic rearrangements [25] and by comparing the genome of SASd strain16-SA00356 to other *S. enterica* subsp. *diarizonae* serovars, we were able to detect large scale genome rearrangements with many inversions as shown in Additional file 3: Figure S2.

## Conclusion

Overall, this study found a number of interesting genomic features linked to pathogenicity and host specificity of SASd to sheep. Among these, we detected increased pseudogene formation, large scale genomic rearrangements, a VirB4/D4 plasmid and novel genomic islands. The complete genome sequence generated in this study forms an important basis for further understanding of the pathogenicity and host adaptation of SASd, as well as a high-quality reference for future genome comparison studies.

## Supplementary information

**Additional file 1: Table S1.** List of *S. enterica* serovars analyzed in this study.

**Additional file 2: Figure S1.** Sequence based similarity of five *S. enterica* subsp. *diarizonae* serovars to *S. enterica* subsp. *diarizonae* serovar 61:k:1,5,(7), isolate 16-SA00356. The sequence similarity is shown by color-coded tracks which from inside to outside represent (i) *S. enterica* subsp. *diarizonae* SA20044251, (ii) *S. enterica* subsp. *diarizonae* NCTC10381, (iii) *S. enterica* subsp. *diarizonae* MZ0080 and (iv) *S. enterica* subsp. *diarizonae* HZS154 and (v) *S. enterica* subsp. d*iarizonae* 11-01853. The location of genetic regions of interest such as Salmonella pathogenicity islands (SPI), genomic islands (GI) and prophage regions are indicated.

**Additional file 3: Figure S2.** Mauve alignment of 16-SA00356, SA20044251, NCTC10381, MZ0080, HZS154 and 11-01853. Colored blocks indicate individual locally collinear blocks (LCB). Homologous LCBs are connected with lines. 16-SA00356 is set as the reference genome.

## Data Availability

Nucleotide sequences were deposited in GenBank under the accession numbers CP034074 (chromosome) and CP034075 (plasmid). The datasets supporting the conclusions of this article are included within the article and its additional files.
