# Supplementary material for: First complete genome sequence and comparative analysis of Salmonella enterica subsp. diarizonae serovar 61:k:1,5,(7) indicates host adaptation traits to sheep
Source: Gut Pathog. 2019 Oct 14;11:48. doi: 10.1186/s13099-019-0330-9 (PMC6791114; doi:10.1186/s13099-019-0330-9)
Supplement: Supplementary file 1 — Additional file 1: Table S1. List of S. enterica serovars analyzed in this study. [file 13099_2019_330_MOESM1_ESM.docx]

**Additional file 1: Table S1: List of *S. enterica* serovars analyzed in this study.**

| **serovar** | **NCBI** | **PubMed** | **Size** | **CDSs** | **tRNA** | **rRNA** | **GC %** |
| --- | --- | --- | --- | --- | --- | --- | --- |
| ***S. enterica* subsp. *diarizonae* serovar 61:-:1,5,7 16-SA00356** | CP034074 | this study | 4832672 bp | 4461 | 83 | 22 | 51.49 |
| *S. enterica* subsp. *diarizonae* serovar 60:r:z 11-01853 | CP011289 | - | 4779348 bp | 4326 | 87 | 22 | 51.51 |
| *S. enterica* subsp. *diarizonae* serovar 60:r:z HZS154 | CP023345 | - | 5087369 bp | 4689 | 91 | 22 | 51.36 |
| *S. enterica* subsp. *diarizonae* serovar 50:k:z MZ0080 | CP022142 | - | 5076950 bp | 4684 | 91 | 22 | 51.54 |
| *S. enterica* subsp. *diarizonae* serovar 61:i:z NCTC10381 | LS483474 | - | 5219421 bp | 4828 | 90 | 22 | 51.21 |
| *S. enterica* subsp. *diarizonae* serovar 65:c:z SA20044251 | CP022135 | - | 4913978 bp | 4461 | 88 | 22 | 51.64 |
| *S. enterica* subsp. *enterica* serovar Agona SL483 | NC_011149 | 21602358 | 4798660 bp | 4444 | 87 | 22 | 52.08 |
| *S. enterica* subsp. *enterica* serovar Choleraesuis SC-B67 | NC_006905 | 15781495 | 4755700 bp | 4566 | 86 | 22 | 52.16 |
| *S. enterica* subsp. *enterica* serovar Dublin CT_02021853 | NC_011205 | 21602358 | 4842908 bp | 4580 | 85 | 22 | 52.18 |
| *S. enterica* subsp. *enterica* serovar Enteritidis P125109 | NC_011294 | 18583645 | 4685848 bp | 4352 | 85 | 22 | 52.17 |
| *S. enterica* subsp. *enterica* serovar Gallinarum 287/91 | NC_011274 | 18583645 | 4658697 bp | 4453 | 77 | 22 | 52.20 |
| *S. enterica* subsp. *enterica* serovar Heidelberg SL476 | NC_011083 | 21602358 | 4888768 bp | 4565 | 84 | 22 | 52.09 |
| *S. enterica* subsp. *enterica* serovar Infantis FSIS1502916 | CP016408 | - | 4728107 bp | 4407 | 86 | 22 | 52.27 |
| *S. enterica* subsp. *enterica* serovar Kentucky SA20030505 | CP022500 | 26941156 | 4782363 bp | 4427 | 87 | 22 | 52.18 |
| *S. enterica* subsp. *enterica* serovar Newport SL254 | NC_011080 | 21602358 | 4827641 bp | 4489 | 88 | 22 | 52.22 |
| *S. enterica* subsp. *enterica* serovar Paratyphi A AKU_12601 | NC_011147 | 19159446 | 4581797 bp | 4340 | 83 | 22 | 52.18 |
| *S. enterica* subsp. *enterica* serovar Paratyphi A ATCC 9150 | NC_006511 | 15531882 | 4585229 bp | 4337 | 83 | 22 | 52.16 |
| *S. enterica* subsp. *enterica* serovar Paratyphi B SPB7 | NC_010102 | - | 4858887 bp | 4549 | 86 | 22 | 52.11 |
| *S. enterica* subsp. *enterica* serovar Paratyphi C RKS4594 | CP000857 | 19229335 | 4833080 bp | 4615 | 84 | 22 | 52.16 |
| *S. enterica* subsp. *enterica* serovar Pullorum ATCC 9120 | CP012347 | 26798102 | 4694842 bp | 4474 | 81 | 23 | 52.19 |
| *S. enterica* subsp. *enterica* serovar Schwarzengrund CVM19633 | NC_011094 | 21602358 | 4709075 bp | 4410 | 84 | 22 | 52.17 |
| *S. enterica* subsp. *enterica* serovar Typhi CT18 | NC_003198 | 11677608 | 4809037 bp | 4665 | 80 | 22 | 52.09 |
| *S. enterica* subsp. *enterica* serovar Typhi Ty2 | NC_004631 | 12644504 | 4791961 bp | 4621 | 80 | 22 | 52.05 |
| *S. enterica* subsp. *enterica* serovar Typhimurium LT2 | NC_003197 | 11677609 | 4857450 bp | 4504 | 86 | 22 | 52.22 |

The tables gives the name of the serovar, NCBI accession number and PubMed accession number (when published). Information to genome size (in bp), number of ORFs, number of tRNAs and rRNAs as well as the GC % content of genomes of the investigated organisms, were obtained through annotation with Prokka (https://github.com/tseemann/prokka) with standard parameters. Only complete genome sequences were considered. Plasmid sequences were excluded from the analysis.
