# Supplementary figures and images for: First complete genome sequence and comparative analysis of Salmonella enterica subsp. diarizonae serovar 61:k:1,5,(7) indicates host adaptation traits to sheep
Source: Gut Pathog. 2019 Oct 14;11:48. doi: 10.1186/s13099-019-0330-9 (PMC6791114; doi:10.1186/s13099-019-0330-9)

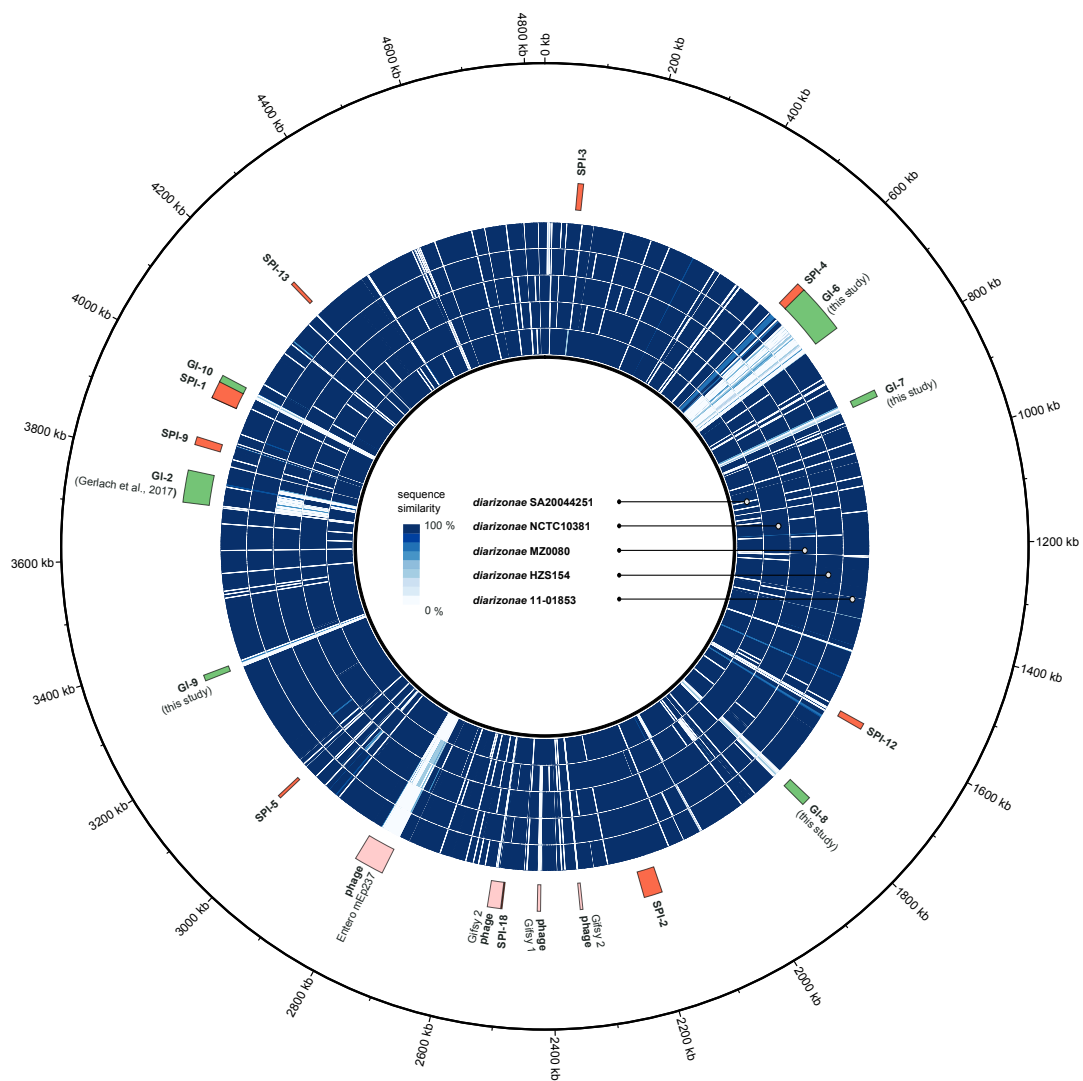

Supplement: Supplementary file 2 — Additional file 2: Figure S1. Sequence based similarity of five S. enterica subsp. diarizonae serovars to S. enterica subsp. diarizonae serovar 61:k:1,5,(7), isolate 16-SA00356. The sequence similarity is shown by color-coded tracks which from inside to outside represent (i) S. enterica subsp. diarizonae SA20044251, (ii) S. enterica subsp. diarizonae NCTC10381, (iii) S. enterica subsp. diarizonae MZ0080 and (iv) S. enterica subsp. diarizonae HZS154 and (v) S. enterica subsp. diarizonae 11-01853. The location of genetic regions of interest such as Salmonella pathogenicity islands (SPI), genomic islands (GI) and prophage regions are indicated. [file 13099_2019_330_MOESM2_ESM.pdf]

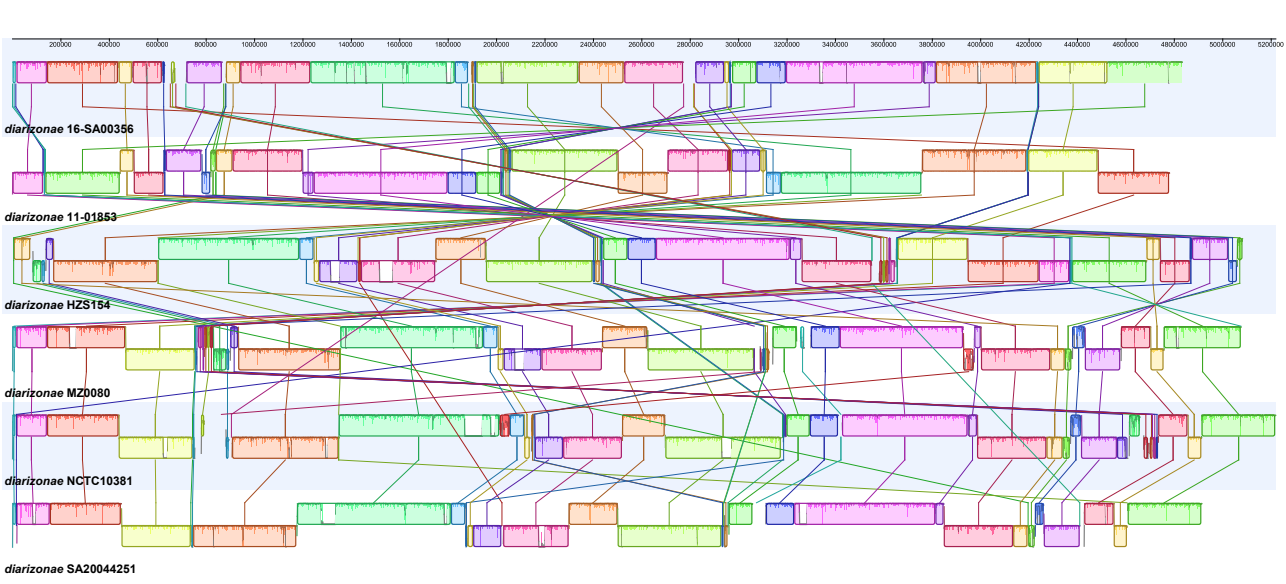

Supplement: Supplementary file 3 — Additional file 3: Figure S2. Mauve alignment of 16-SA00356, SA20044251, NCTC10381, MZ0080, HZS154 and 11-01853. Colored blocks indicate individual locally collinear blocks (LCB). Homologous LCBs are connected with lines. 16-SA00356 is set as the reference genome. [file 13099_2019_330_MOESM3_ESM.pdf]
